# Body fluids and salt metabolism - Part I

**DOI:** 10.1186/1824-7288-35-36

**Published:** 2009-11-19

**Authors:** Mario G Bianchetti, Giacomo D Simonetti, Alberto Bettinelli

**Affiliations:** 1 Department of Pediatrics, Bellinzona and Mendrisio and University of Bern, Bern, Switzerland; 2 Pediatric Nephrology, University Children's Hospital Bern and University of Bern, Bern, Switzerland; 3 Department of Pediatrics, San Leopoldo Mandic Hospital, Merate-Lecco, Italy

## Abstract

There is a high frequency of diarrhea and vomiting in childhood. As a consequence the focus of the present review is to recognize the different body fluid compartments, to clinically assess the degree of dehydration, to know how the equilibrium between extracellular fluid and intracellular fluid is maintained, to calculate the effective blood osmolality and discuss both parenteral fluid requirments and repair.

## Introduction

### Body fluid compartments

**Figure 1:**
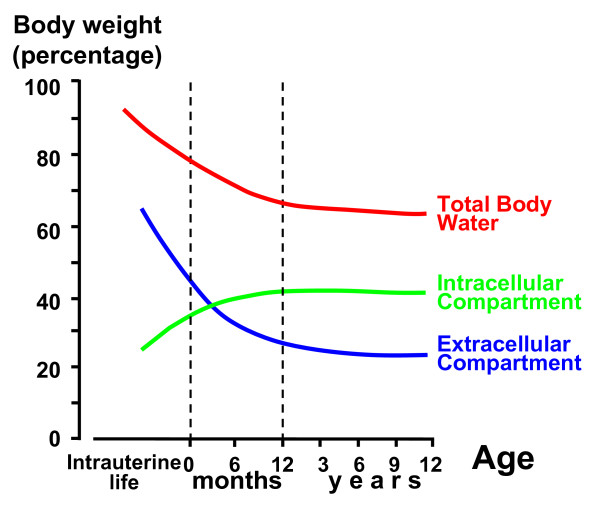
**Winters diagram with the subdivision of total body water, intracellular fluid and extracellular fluid as a function of age**. For clinical purpose the use of "the rule of 3" is recommended: 1. total body water makes up 2/3 of the body mass; 2. the intracellular compartment contains 2/3 of the total body water and the remaining (= 1/3) is held in the extracellular compartment; 3. the extracellular compartment is further subdivided into the interstitial and the intravascular compartments (blood volume), which contain 2/3 and 1/3 of the extracellular fluid, respectively. After puberty males generally have 2 to 10 percent higher water content than females.

Water makes up 50-75 percent of the body mass. The most important determinants of the wide range in water content are age and gender: a. the water content of a newborn, an adolescent and an elderly man are approximately 75, 60 and 50 percent; b. after puberty males generally have 2 to 10 percent higher water content than females (figure 1). The intracellular compartment contains about two-third of the total body water and the remaining is held in the extracellular compartment. The solute composition of the intracellular and extracellular fluid differs considerably because the sodium pump maintains potassium in a primarily intracellular and sodium in a primarily extracellular location. Consequently potassium largely determines the intracellular and sodium the extracellular compartment [1-3]. The extracellular compartment is further subdivided into the interstitial and the intravascular compartments (blood volume), which contain two-thirds and one-third of the extracellular fluid, respectively. Finally, the transcellular fluid compartment comprises the digestive, cerebrospinal, intraocular, pleural, peritoneal and synovial fluids but will not be further addressed in this review.

**Figure 2:**
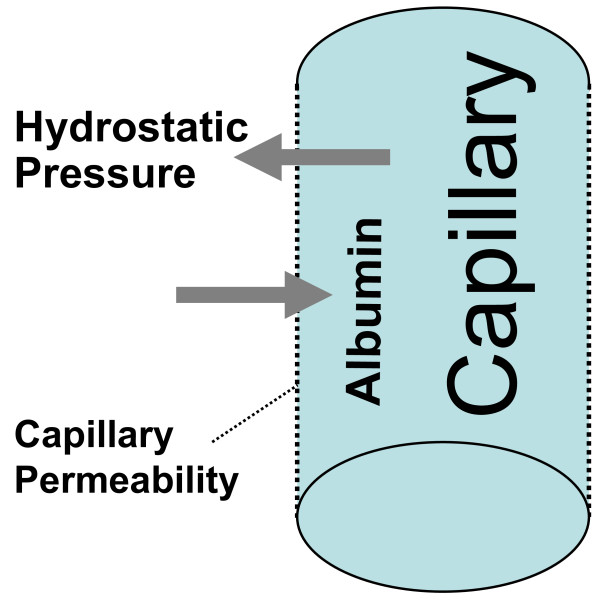
**Distribution of ultrafiltrate across the capillary membrane**. The barrel-shaped structure represents a capillary. A high hydrostatic pressure or an increased capillary permeability causes fluid to leave the vascular space. On the contrary an increased intravascular albumin concentration and, therefore, an increased oncotic pressure causes fluid to enter the vascular space.

**Figure 3:**
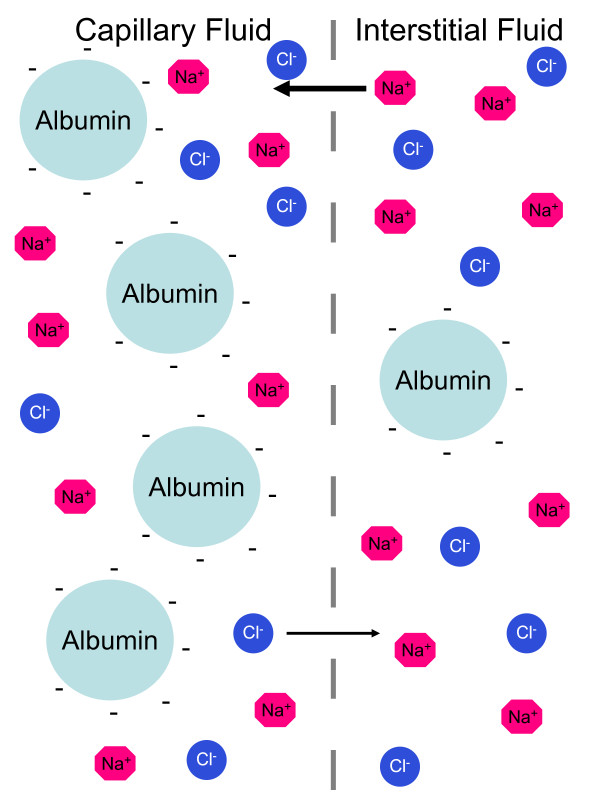
**The Gibbs-Donnan effect**. There is a different concentration in the concentration of anionic albumin, which is impermeant, between the vascular (albumin approximately 40 g/L) and the interstitial (albumin approximately 10 g/L) compartments. The negative charges of albumin "attract" cations (largely Na^+^) into the vascular compartment and "repell" anions (Cl^- ^and HCO_3 _^-^) out. Because the concentration of Na^+^exceeds that of Cl^- ^and HCO_3_^-^, "attraction" outweighs "repulsion". Consequently the Gibbs-Donnan effect increases the vascular compartment. The dashed line represents the capillary bed separating the intravascular and interstitial spaces is freely permeable to Na^+^, K^+^, Cl^-^, and glucose.

The size of the intravascular compartment is determined by the overall size of the extracellular fluid compartment and by the Starling forces: they control the partition of fluids between intravascular and interstitial compartments across the capillary membrane that is crossed by salts like sodium chloride and by glucose but not by blood proteins (especially albumin). Three major forces control the distribution of fluids across the capillary membrane (figure 2): a. the hydrostatic pressure causes fluids to leave the vascular space, and; b. the higher concentration of proteins in the intravascular compartment as compared with that in interstitial fluid, which causes fluids to enter the vascular space. This force, which is called oncotic pressure, is due both to the concentration gradient of albumin (blood proteins other than albumin account for 50 percent of the weight of proteins in g in blood but only for 25 percent of the oncotic pressure) as well to the fact that albumin is anionic and therefore attracts cations (largely sodium) into the vascular compartment (Gibbs-Donnan effect; figure 3). c. Capillary permeability is a further major mechanism that modulates the distribution of fluids across the capillary membrane.

### Effective circulating volume

Effective circulating volume denotes the part of the intravascular compartment that is in the arterial system and is effectively perfusing the tissues. The effective circulating volume is biologically more relevant than the intravascular compartment and usually varies directly with the extracellular fluid volume [4]. As a result, the regulation of extracellular fluid balance (by alterations in urinary sodium excretion) and the maintenance of the effective circulating volume are intimately related. Sodium loading will tend to produce volume expansion, whereas sodium loss (e.g., due to vomiting, diarrhea, or drug management with diuretics) will lead to volume depletion. The body responds to changes in effective circulating volume in two steps: 1. The change is sensed by the volume receptors, which are located in the cardiopulmonary circulation, the carotid sinuses and aortic arch, and in the kidney; 2. These receptors activate effectors that restore normovolemia by varying vascular resistance, cardiac output, and renal water and salt excretion. Briefly, the extrarenal receptors primarily govern the activity of the sympathetic nervous system and natriuretic peptides. On the other side the renal receptors affect volume balance by modulating the renin-angiotensin II-aldosterone system.

In some settings the effective circulating volume is independent of the extracellular fluid volume. Among patients with heart failure the extracellular fluid volume is increased but the patient is effectively volume depleted due to the low cardiac output.

### Blood osmolality - measurement of sodium

Osmolality is the concentration of all of the solutes in a given weight of water. The total (or true) blood osmolality is equal to the sum of the osmolalities of the individual solutes in blood. Most of the osmoles in blood are sodium salts, with lesser contributions from other ions, glucose, and urea. However, under normal circumstances, the osmotic effect of the ions in blood can usually be estimated from two times the sodium concentration. Blood osmolality (in mosm/kg H_2_0) can be measured directly (via determination of freezing point depression) or estimated from circulating sodium, glucose and urea (in mmol/L [To obtain glucose in mmol/L divide glucose in mg/dL by 18. To obtain urea in mmol/L divide urea nitrogen in mg/dL by 2.8 or urea in mg/dL by 6.0.]) as [5-9]:

The effective blood osmolality, known colloquially as blood tonicity, is a further clinically significant entity, which denotes the concentration of solutes impermeable to cell membranes (sodium, glucose [Glucose is a unique solute because, at normal concentrations in blood, it is actively taken up by cells and therefore acts as an ineffective solute, but under conditions of impaired cellular uptake (like diabetes mellitus) it becomes an effective extracellular solute.], mannitol) and are therefore restricted to the extracellular compartment (osmoreceptors sense effective blood osmolality rather than the total blood osmolality). These solutes are effective because they create osmotic pressure gradients across cell membranes leading to movement of water from the intracellular to the extracellular compartment. Solutes that are permeable to cell membranes (urea, ethanol, methanol) are ineffective solutes because they do not create osmotic pressure gradients across cell membranes and therefore are not associated with such water shifts. Since no direct measurement of effective blood osmolality (which is biologically more important than the total or true blood osmolality) is possible, following equations are used to calculate this entity:

Flame photometry, the traditional assay for circulating sodium, measures the concentration of sodium per unit volume of solution, with a normal range between 135 and 145 mmol/L. In fact, sodium is dissolved in plasma water, which normally accounts for 93% of the total volume of plasma, the remaining 7% consisting of protein and lipid. Ion selective electrodes, that have replaced flame photometry in most laboratories, determine the activity of sodium in plasma water, which ranges between 145 and 155 mmol/L. For convenience, laboratories routinely apply a correction factor so that the reported values still correspond to the traditional normal range of 135-145 mmol/L [5-9]. A kind of "pseudohyponatraemia" caused by expansion of the non-aqueous phase of plasma - for example, due to hyperlipidaemia or paraproteinemia - is no longer seen because determination by selective electrodes in undiluted serum, plasma or whole blood is unaffected by this [The recommended name for this quantity is ionized sodium.] [9]. Although, strictly speaking, a sodium concentration outside the range of 135-145 mmol/L denotes dysnatremia, clinically relevant hypo- or hypernatremia is mostly defined as a sodium concentration outside the extended normal range of 130-150 mmol/L [5-9].

### Dehydration and extracellular fluid volume depletion

The terms extracellular fluid volume depletion and dehydration are mostly used interchangeably. However, these terms denote conditions resulting from different types of fluid losses. Volume depletion refers to any condition in which the effective circulating volume is reduced. It is produced by salt and water loss (as with vomiting, diarrhea, diuretics, bleeding, or third space sequestration). Strict sense dehydration refers to water loss alone. The consequence of strict sense dehydration is hypernatremia. The elevation in serum sodium concentration, and therefore effective blood osmolality, pulls water out of the cells into the extracellular fluid. However, much of the literature does not distinguish between the two terms. Although dehydration in general usage means loss of water, in physiology and medicine, the word means both a loss of water and salt. Depending on the type of pathophysiologic process, water and sodium may be lost in physiologic proportion or lost disparately, with each type producing a somewhat different clinical picture, designated as normotonic (mostly isonatremic), hypertonic (mostly hypernatremic), or hypotonic (always hyponatremic) dehydration. Dehydration develops when fluids are lost from the extracellular space at a rate exceeding intake. The most common sites for fluid loss are 1. the intestinal tract (diarrhea, vomiting, or bleeding), 2. the skin (fever, burns, or cystic fibrosis) and 3. the urine (osmotic diuresis, diuretic therapy, or diabetes insipidus). More rarely, dehydration results from prolonged inadequate intake without excessive losses [1-3,10].

Children and especially infants are more susceptible to dehydration than adults. The risk is high for the following causes: a. infants and children are more susceptible to infectious diarrhea and vomiting than adults; b. there is a higher proportional turnover of body fluid in infants compared to adults (it is estimated that the daily fluid intake and outgo, as a propotion of extracellular fluid, is in infancy twice that of an adult); c. young children do not communicate their need for fluids or do not independently access fluids to replenish volume losses [1-3,10].

Dehydration reduces the effective circulating volume, therefore impairing tissue perfusion. If not rapidly corrected, ischemic end-organ damage occurs, leading to serious morbidity.

Three groups of symptoms and signs occur in dehydration [1-3,5-10]: a. those related to the manner in which fluids loss occurs (including diarrhea, vomiting or polyuria); b. those related to the electrolyte and acid-base imbalances that sometimes accompany dehydration; and c. those directly due to dehydration. The following discussion will focus the third group.

When assessing a child with a tendency towards dehydration, the clinician needs to address the degree of extracellular fluid volume depletion. More rarely the clinician will address the laboratory testing and the type of fluid lost (extracellular or intracellular fluid).

#### Degree of dehydration

It is imperative to accurately assess the degree of dehydration since severe extracellular fluid volume depletion calls for rapid fluid resuscitation [10,11]. Dehydration is most objectively measured as a change in weight from baseline (acute loss of body weight reflects the loss of fluid, not lean or fat body mass; thus, a 1.2 kg weight loss should reflect the loss of 1.2 liters of fluid). In most cases, however, a previous recent weight is unavailable.

As a result, a pertinent history and a number of findings on physical examination are used to help assess dehydration. Skin turgor, sometimes referred to as skin elasticity, is a sign commonly used to assess the degree of hydration. The skin on the back of the hand, lower arm, or abdomen is grasped between two fingers, is held for a few seconds then released: skin with normal turgor snaps rapidly back to its normal position but skin with decreased turgor remains elevated and returns slowly to its normal position. However, decreased skin turgor is a late sign in dehydration that is associated with moderate or, more frequently, severe dehydration. Like decreased skin turgor, arterial hypotension is a late sign in hypovolemia that is rapidly followed by cardiac arrest (in children with minimal to mild dehydration blood pressure is often slightly increased). Symptoms and signs of dehydration include dry mucous membranes, sunken eyes, reduced urine output, a sunken open fontanelle, delayed capillary refill, deep respiration with or without increased respiratory rate, and tachycardia. Several attempts have been made to determine a measure of dehydration by using combinations of clinical findings. Very recently, in children < 4 years of age with a diagnosis of acute gastroenteritis, 4 clinical items (a. general appearance, b. eyes, c. mucous membranes, and d. tears), which may be summed for a total score ranging from 0 to 8 [12], were found to significantly estimate dehydration (table 1).

**Table 1:** "4-item 8-point rating scale" clinical dehydration scale [12].

	Score		
**Characteristic**	**0**	**1**	**2**

General appearance	Normal	Thirsty, restless or lethargic but irritable when touched	Drowsy, limp, cold, or sweathy; comatose or not
Eyes	Normal	Slightly sunken	Very sunken
Mucous membranes (tongue)	Moist	Sticky	Dry
Tears	Tears	Decreased tears	Absent tears

The score consists of 4 clinical items, which may be summed for a total score ranging from 0 to 8. The final 3 categories are no or minimal dehydration (< 3%; score of 0), mild dehydration (3% to < 6% dehydration; score of 1--4), and moderate to severe dehydration (≥ 10% dehydration; score of 5--8).

#### Laboratory testing and the type of fluid lost

Laboratory testing can confirm the presence of dehydration [13]. The fractional excretion of sodium (which measures the amount of filtered sodium that is excreted in the urine):

is < 0.5 × 10^-2 ^(< 0.5 percent) and the urine spot sodium concentration < 30 mmol/L (unless the source of dehydration is renal).

Furthermore, in dehydration, the urine is concentrated with an osmolality > 450 mosm/kg H_2_O. The urinary concentration can be measured with an osmometer or fairly estimated, in the absence of proteinuria and glucosuria, from the specific gravity, as determined by refractometry, as follows:

Dipstick measurement of specific gravity is very popular but unfortunately unreliable [14].

Furthermore, laboratory testing can detect associated electrolyte and acid-base disturbances but determination of circulating electrolytes and acid-base balance is typically limited to children requiring intravenous fluids. These children are more severely volume depleted and are therefore at greater risk for dyselectrolytemias. Laboratory testing is less useful for assessing the degree of volume depletion.

**Figure 4:**
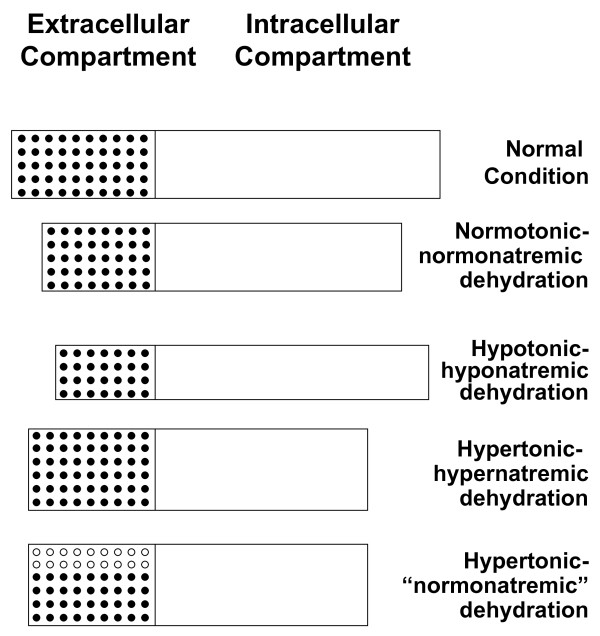
**Extracellular and intracellular compartments in children with dehydration**. Normally the extracellular compartment makes up approximately 20 percent and the intracellular 40 percent of the body weight (upper panel of the figure). The second, third and fourth panels depict the relationship between extracellular and intracellular compartment in three children with dehydration in the context of an acute diarrheal disease: dehydration is normotonic-normonatremic in the first, hypotonic-hyponatremic (mainly extracellular fluid losses) in the second, and hypernatremic (mainly intracellular fluid losses) in the third child. The lower panel depicts the relationship between extracellular and intracellular compartment (mainly intracellular fluid losses) in a child with dehydration in the context of diabetic ketoacidosis (hypertonic-"normonatremic" dehydration; in this context the concentration of circulating sodium is normal or even reduced). In each panel the solid circles denote sodium and open circles impermeable solutes that do not move freely across cell membranes (in the present example glucose). For reasons of simplicity, no symbols are given for potassium, the main intracellular cation.

- Bicarbonatemia ≤ 17.0 mmol/L might be the most useful laboratory test to assess dehydration. The blood urea level reflects the severity of dehydration, the decreased glomerular filtration rate and the increased sodium and water reabsorption in the proximal tubule [11]. Unfortunately the clinical usefulness of this test is limited, since this blood parameter can be increased by other factors such as bleeding or tissue breakdown (on the other side the rise can be minimized by a concurrent decrease in protein intake).

- The sodium concentration varies with the relative loss of solute to water. Changes in sodium concentration play a pivotal role in deciding the type of fluid depletion (figure 4):

Hyponatremic and hypotonic dehydration [5,6]: The development of hyponatremia reflects net solute loss in excess of water loss. This does not occur directly, as fluid losses such as diarrhea are not hypertonic. Usually solute and water are lost in proportion, but water is taken in and retained in the context of hypovolemia-induced secretion of antidiuretic hormone. Since body water shifts from extracellular fluid to cells under these circumstances, signs of dehydration easily become profound.

Normonatremic and isotonic dehydration: In this setting, solute is lost in proportion to water loss.

Hypernatremic and hypertonic dehydration [7,8]: This setting reflects water loss in excess of solute loss. Since body water shifts from intracellular to extracellular fluid under these circumstances, these children have less signs of dehydration for any given amount of fluid loss than do children with normonatremic (or normotonic) dehydration and especially those with hyponatremic dehydration.

## Conclusion

Clinical assessment of dehydration may be difficult, especially in young infants. A large body of evidence suggests the use of a recently developped and validated "4-item 8-point rating scale". Mainly extracellular fluid losses occur in hypotonic dehydration, where signs of dehydration easily become profound; on the contrary, mainly intracellular fluid losses occur in hypertonic dehydration, where signs of dehydration tend to be less evident.

## Competing interests

The authors declare that they have no competing interests.

## Authors' contributions

MGB and AB wrote the first version of the manuscript. GDS revised the manuscript and prepared the figures. All authors have read and approved the paper, have met the criteria for authorship as established by the International Committee of Medical Journals Editors, believe that the paper represents honest work, and are able to verify the validity of the results reported.
